# A Generalized Measurement Model to Quantify Health: The Multi-Attribute Preference Response Model

**DOI:** 10.1371/journal.pone.0079494

**Published:** 2013-11-21

**Authors:** Paul F. M. Krabbe

**Affiliations:** 1 University of Groningen, University Medical Center Groningen, Department of Epidemiology, Groningen, The Netherlands; 2 Theta Research, Zeist, The Netherlands; University of Otago, New Zealand

## Abstract

After 40 years of deriving metric values for health status or health-related quality of life, the effective quantification of subjective health outcomes is still a challenge. Here, two of the best measurement tools, the discrete choice and the Rasch model, are combined to create a new model for deriving health values. First, existing techniques to value health states are briefly discussed followed by a reflection on the recent revival of interest in patients’ experience with regard to their possible role in health measurement. Subsequently, three basic principles for valid health measurement are reviewed, namely unidimensionality, interval level, and invariance. In the main section, the basic operation of measurement is then discussed in the framework of probabilistic discrete choice analysis (random utility model) and the psychometric Rasch model. It is then shown how combining the main features of these two models yields an integrated measurement model, called the multi-attribute preference response (MAPR) model, which is introduced here. This new model transforms subjective individual rank data into a metric scale using responses from patients who have experienced certain health states. Its measurement mechanism largely prevents biases such as adaptation and coping. Several extensions of the MAPR model are presented. The MAPR model can be applied to a wide range of research problems. If extended with the self-selection of relevant health domains for the individual patient, this model will be more valid than existing valuation techniques.

## Introduction

The measurement of health, which is defined as assigning meaningful numbers to an individual’s health status, has proliferated ever since the World Health Organization (WHO) provided its definition of health in 1946 [1]. It wasn’t until 1970 that Fanshel and Bush introduced the first instrument that was able to capture an individual’s health state in a single metric value [2]. Access to single metric values for health states is advantageous as these can be used in health outcomes research, disease modeling studies, economic evaluations, and to monitor the health status of patient groups in the general community. Often the values for health states are expanded by combining it with the duration of these states to obtain health summary measures. A well known example of such a summary measure is the disability-adjusted life years (DALYs) approach that is being used by the WHO to compare different countries with one another on diverse aspects of health. Health economists often apply a rather comparable health summary measure, namely the quality-adjusted life year (QALY).

To quantify health states, these must be described and classified in terms of seriousness and assigned meaningful values (variously called utilities, strength of preference, index, or weights). The first step is thus to clarify the concept of health status. Essentially an umbrella concept, it covers independent health domains that together capture the not yet well defined notion of health-related quality of life (HRQoL). The second step is to assign a value to the health-state description by means of an appropriate measurement procedure. In the past, several measurement models have been developed to quantify subjective phenomena and some of these models have found their way into the valuation of health states. Although the scientific enterprise of measuring health states has been going on for about 40 years, there are still concerns about validity.

The aim of this paper is to forge a linkage between two prominent measurement models to create a single general model that – at least in principle – resolves many of the problems posed by widely used but inferior valuation techniques. This new measurement framework for deriving health-state values is called the multi-attribute preference response (MAPR) model. It combines the characteristics of hypothetical health states with a respondent’s health-status characteristics to quantify both the hypothetical states as well as the location of the patients’ s state. In theory, this new model even allows individuals to choose the attributes (i.e., health domains) describing their health states. A health measurement model with such potential flexibility is unprecedented.

The first section of the paper presents some concerns about the validity of current health-state valuation techniques followed by a section about the basic measurement principles of subjective phenomena (such a health). The next section explains the probabilistic discrete choice model and expands on its relationship to measurement models used in economics and psychology. The subsequent section sketches the history of the Rasch model and summarizes its underlying theory. Finally, the merits of integrating these two measurement models into the MAPR model are discussed. All examples and suggestions in this article apply to health-state valuation. It should be kept in mind that because the MAPR model is very general, it can also be applied in a number of other fields where the goal is to quantify other subjective phenomena.

## Existing Valuation Techniques

The standard gamble (SG) and time trade-off (TTO) are frequently used to assign values to health states. The former emerged from the field of economics, the latter from the area of operations research [3], [4]. SG, for years the gold standard, was developed under the expected utility theory of von Neumann and Morgenstern [3]. But as experience shows, assumptions underlying this theory were systematically violated by human behavior. In general, people have difficulty dealing with probabilities and may have an aversion to taking risk. As an alternative, Torrance and colleagues developed TTO, which is simpler to administer than SG. The main drawback of TTO is that the relationship between a health state, its duration, and its value is collapsed into a single measure. The problem is that this requires the values for health states to be independent of the duration of these states. Health-state values have also been derived by another technique, the visual analogue scale (VAS), which stems from the field of psychology [5]. Unfortunately, all of these conventional measurement techniques (SG, TTO, VAS) have theoretical and empirical drawbacks when used to value health states. With the possible exception of the VAS, they put a large cognitive burden on the respondents by demanding a relatively high degree of abstract reasoning [6]. The person trade-off (PTO) is another technique that has been used mainly in the area of policy making [7]. This technique was named by Nord [8], but the technique itself was applied earlier by Patrick et al. [9]. The PTO asks respondents to answer from the perspective of a social decision-maker considering alternative policy choices.

The currently dominant valuation technique for quantifying health states, certainly in the field of health economics, is the time trade-off (TTO). It may be intuitively appealing for three reasons. First, it seems to reflect the actual medical situation. Second, it shows some correspondence to the general health-outcome framework (since the TTO is essentially a QALY equivalence statement). And third, it is grounded in economic thinking (the trade-off principle). Nevertheless, compelling arguments against the TTO have been raised by several authors [10], [11], [12], [13], [14]. In fact, TTO seems to be associated with many problems: practical (difficult for people to perform), theoretical (axiomatic violations, problems in dealing with states worse than dead), and biases (time preference). From a measurement perspective, the TTO technique has been criticized for its susceptibility to framing issues (e.g., duration of the time frame, indifference procedure, states worse than dead). The same holds for the recently introduced technique known as lead-time TTO [15].

### Patients versus General Population

Conventionally, values for the health states used in economic evaluations are derived from a representative community sample [16], or in the case of the DALY approach, values for disease states were derived from medical experts [17]. Besides asserting that a sample of the general population is a reflection of the average taxpayer, which is considered fair grounds for arriving at resource allocation, other arguments are put forward. For example, it has been noted that patients may adapt to their health state over a period of time. As a result, they may assign higher values to their own poor health state. Patients may also strategically underrate the quality of their health state, knowing they will directly benefit from doing so (e.g., certain patient groups may be considered as more relevant by policy makers, or effects in cost-effectiveness studies may show more favorable results). The proposition held in this paper is that while adaptation is a real phenomenon, this effect can largely be reduced and eventually eliminated if the health-state values are derived in a fitting measurement framework. Moreover, it is reasonable to assume that healthy people may be inadequately informed or lack the imagination to make an appropriate judgment on the impact of severe health states. This is one of the reasons why researchers in the field of HRQoL are engaged in a debate about which values are more valid [18], [19]. Many researchers assert that individuals are the best judges of their own health status [20]. Therefore, in a health-care context, it is sensible to defend the position that, from a validity perspective, it is the patient’s judgment that should be elicited in order to arrive at health-state values, not that of a sample of unaffected members of the general population. This explains the rise of the so-called patient-reported outcome measurement (PROMs) movement [21]. Voices from another area have also stressed that such assessments from patients (experienced utility) should get more attention [22], [23].

## Measurement Principles

### Interval Level

There are theoretical and methodological differences between the direct valuation techniques (SG, TTO, VAS) and indirect (latent) measurement models such as probabilistic discrete choice (DC; see next section). But they all assume that individuals possess implicit preferences for health states that range from good to bad. And all of the models maintain that it should be possible to reveal these preferences and express them quantitatively. Accordingly, differences between health states should reflect the increments of difference in severity of these states. For that reason, informative (i.e., metric) outcome measures should be at least at the interval level (cardinal data). This means that measures should lie on a continuous scale, whereby the differences between values would reflect true differences (e.g., if a patient’s score increases from 40 to 60, this increase is the same as from 70 to 90). To arrive at health-state values with these qualities, two other basic measurement principles should be fulfilled, namely unidimensionality and invariance.

### Unidimensionality

The overall goal is to use health-state values for computational procedures (e.g., computing QALYs, Markov modeling). For that reason, informative (i.e., metric) outcome measures should be at least at the interval level. This implies positioning the values on an underlying unidimensional scale ranging from the worst health state to the best one. An (implicit) assumption made in the field of health-state valuation is that, in general, individuals evaluate health states similarly, which permits the aggregation of individual valuations to arrive at group or societal values. Specific analyses can be applied to find empirical evidence that health-state values represent a unidimensional structure. An early application of the statistical singular value decomposition routine compared TTO and VAS valuation data. The results showed a clear two-dimensional structure for the TTO [24]. Heterogonous responses (or even distinct response structures) by individuals may indicate that the phenomenon under study (health states) is not characterized as unidimensional or that a certain valuation technique is less appropriate for the task, since it may not fulfill the need for unidimensional responses. Therefore, it is important to determine how similar individuals’ judgments (inter-rater reliability) actually are.

### Invariance

Invariance is a critical prerequisite for fundamental measurement (see section: Rasch model). It means that the outcome of judgments between two (or more) health states should not dependent on which group of respondents performed the assessments. The resulting judgments among health states should also be independent of the set of health states being assessed [25]. In the setting of health-state valuation the invariance principle appears to be closely related to the unidimensionality requirement. Rasch models embody the invariance principle. Their formal structure permits algebraic separation of the person and health-state parameters. Specifically, the person parameter can be eliminated during the process of statistical estimation of the health-state parameters. Not surprisingly, the invariance principle is a key characteristic of measurement in physics [25].

## Discrete Choice Model

### Background

Modern probabilistic discrete choice (DC) models, which come from econometrics, build upon the work of McFadden, the 2000 Nobel Prize laureate in economics [26]. DC models encompass a variety of experimental design techniques, data collection procedures, and statistical procedures that can be used to predict the choices that individuals will make between alternatives (e.g., health states). These techniques are applicable when individuals have the ability to choose between two or more distinct (‘discrete’) alternatives.

In the mid-1960s McFadden was working with a graduate student, Phoebe Cottingham, trying to analyze data on freeway routing decisions as a way to study economic decision-making behavior. He developed the first version of what he called the ‘conditional multinomial logistic model’ (also known as the multinomial logistic model and conditional logistic model). McFadden proposed an econometric model in which the utilities of alternatives depend on utilities assigned to their attributes, such as construction cost, route length, and areas of parkland and open space taken up [27]. He developed a computer program that allowed him to estimate this probabilistic model, which was based on an axiomatic theory of choice behavior developed by the mathematical psychologist Luce [28].

The DC strategy was conceived in transport economics and later disseminated into other research fields, especially marketing. There, DC modeling was applied to analyze behavior that could be observed in real market contexts. Instead of modeling the choices people actually make in empirical settings, Louviere and others started to model the choices made by individuals in carefully constructed experimental studies [29]. This entailed presenting the participants with profiles containing features of hypothetical products. Originally, these profiles were known as simulated choice situations, but later they were called discrete choice experiments (DCEs). So, instead of modeling actual choices, as McFadden had with the revealed preferences approach, Louviere modeled choices made in experimental studies with the stated preferences approach. This new approach also made it possible to predict values for alternatives that could not be judged in the real world. More recently, DC models have been used as an alternative way to derive people’s values for health states [30], [31], [32].

### Measurement Model

The statistical literature classifies DC models among the probabilistic choice models that are grounded in modern measurement theory and consistent with economic theory (e.g., the random utility model) [33]. What all DC models have in common is that they can establish the relative merit of one phenomenon with respect to others. If the phenomena are characterized by specific attributes or domains with certain levels, extended DC models such as McFaddens’ model would permit estimating the relative importance of the attributes and their associated levels. DC modeling has good prospects for health-state valuation [32], [34], [35], [36], [37], [38]. Moreover, DC models have a practical advantage: when conducting DCEs, health states may be evaluated in a self-completion format. The scope for valuation research is thereby widened. Most TTO protocols for deriving values for preference-based health-state instruments are interviewer-assisted, as studies have clearly showed that self-completion is not feasible or leads to inaccurate results [39]. The simplicity of DC tasks, however, facilitates web-based surveys [38].

#### Discrimination mechanism

The modern measurement theory inherent in DC models builds upon the early work and basic principles of Thurstone’s Law of Comparative Judgment (LCJ) [40], [41]. In fact, the class of choice- and rank-based models, with its lengthy history (1927 to the present), is one of the few areas in the social and behavioral sciences that has a strong underlying theory. It was Thurstone who introduced the well-known random utility model (RUM), although he used different notation and other terminology. The use of Thurstone’s model based on paired comparisons to estimate health-state values was first proposed by Fanshel and Bush [2] in one of the earliest examples of a composed QALY index model.

In Thurstone’s terminology, choices are mediated by a ‘discriminal process’. He defined this as the process by which an organism identifies, distinguishes, or reacts to stimuli. Consider the theoretical distributions of the discriminal process for any two objects (paired comparisons), like two different health states *s* and *t*. In the LCJ model, the standard deviation of the distribution associated with a given health state is called the discriminal dispersion (or variance, in modern scientific language) of that health state. Discriminal dispersions may differ for different health states.

Let *v_s_* and *v_t_* correspond to the scale values of the two health states. The difference (*v_s_–v_t_*) is measured in units of discriminal differences. The complete form of the LCJ is the following equation.
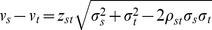
(1)where *σ*_s_, *σ*_t_ denotes the discriminal dispersions of the two health states *s* and *t*, 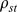 denotes the correlation between the pairs of discriminal processes *s* and *t*, and 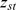 is the unit normal deviate corresponding to the theoretical proportion of times health state *s* is judged greater than health state *t*. The difference is normally distributed with mean *v_s_ – v_t_* and variance 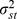 corresponding to 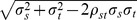, which reflects the standard deviation of the difference between two normal distributions. In its most basic form (Case V) the model can be represented as 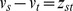, for which the probability that state *s* is judged to be better than state *t* is.
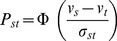
(2)where Φ is the cumulative normal distribution with mean zero and variance unity.

The discrimination mechanism underlying the LCJ is an extension of the ‘just noticeable difference’ that played a major role in early psychophysical research, as initiated by Fechner (1801–1887) and Weber (1795–1878) in Germany. Later on similar discrimination mechanisms were embedded in ‘signal detection theory’, which was used by psychologists to measure the way people make decisions under conditions of uncertainty. Much of the early work in this research field was done by radar researchers [42].

#### Random utility model

Thurstone proposed that perceived physical phenomena or subjective concepts (e.g., health states, treatment outcomes, process characteristics) can be expressed as that a respondent *r* has a latent value (utility) for state *s*, *U_rs_*, which includes a systematic component and an error term (This is equal to the fundamental idea of true score theory or classical test theory. The latter also consists of an observed score with two components, namely the true score and an error term. It too summarizes different health domains by combining the scores on several items.):
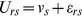
(3)


Here, *v* is the measurable component and is not determined by characteristics of the respondents. In other words, a given health state has the same expected value across all respondents. The assumption in the model proposed by Thurstone is that *ε* is normally distributed. This assumption yields the probit model. The choice probability is *P_rs_* = Pr(*U_rs_>U_rt_*, all *t* not equal to *s*), which depends on the difference in value, not on its absolute level. The fact that only differences in value matter has implications for the identification of this model and all its derivates. In particular, it means that the only parameters that can be estimated are those that capture differences across alternatives.

So, in Thurstone’s LCJ, the perceived value of a health state equals its objective level plus a random error. The probability that one health state is judged better than another is the probability that this alternative has the higher perceived value. When the perceived values are interpreted as levels of satisfaction, HRQoL, or utility, this can be interpreted as a model for economic choice in which utility is modeled as a random variable. This assertion was made in 1960 by the economist Marschak, who thereby introduced Thurstone’s work into economics. Marschak called his model the random utility maximization hypothesis or RUM [43], [44]. Like neoclassical economic theory, the RUM assumes that the decision-maker has a perfect discrimination capability. But it also assumes that the analyst has incomplete information, which implies that uncertainty (i.e., randomness) must be taken into account.

#### Multinomial model

Another way to analyze comparative data is with the Bradley-Terry-Luce (BTL) model, which was statistically formulated by Bradley and Terry in 1955 [45] and extended by Luce in 1959 [28] (Later it was recognized that the German mathematician Ernst Zermelo had already published about a probabilistic paired comparison model [46]). The BTL models extends the Thurstone model by allowing a person to choose among more than two options. It postulates that measurement on a ratio scale level can be established if the data satisfy certain structural assumptions [47]. For mathematical reasons the BTL model is based on the simple logistic function instead of the normal distribution of the Thurstone model. It is this mathematical model that McFadden used to develop and construct his own specific type of multinomial logit model. If only pairs of alternatives are judged, the BTL model is nearly identical to Thurstone’s model. However, when more than two alternatives are judged, an important mathematical assumption must be made, namely the independence of irrelevant alternatives (see below).

Drawing upon the work of Thurstone, Luce, Marschak, and Lancaster [48], McFadden was able to show how his model fit in with the economic theory of choice behavior. McFadden then investigated further the RUM foundations of the conditional multinomial logistic model. He showed that the Luce model was consistent with the RUM model with IID (independent and identically distributed random variables) additive disturbances if and only if these disturbances had a distribution called extreme value type I. More importantly, instead of one function, as in the classical Thurstone model (only values for health states can be estimated), the conditional multinomial logistic model comprises two functions. First, it contains a statistical model that describes the probability of ranking a particular health state higher than another, given the (unobserved) value associated with each health state. Secondly, it contains a valuation function that relates the value for a given health state to a set of explanatory variables (it will be shown that the same holds for the MAPR model).

#### Assumptions

Multinomial logistic regression (MNL) is based on three assumptions: (*i*) independence of irrelevant alternatives (IIA); (*ii*) error terms are independent and identically distributed across observations (IID); and (*iii*) no taste heterogeneity (i.e., homogeneous preferences across respondents). Luce’s choice axiom states that the probability of selecting one item over another from a pool of many items is not affected by the presence or absence of other items in the pool (IIA assumption). The axiom states that if A is preferred to B out of the choice set {A, B}, then introducing a third, irrelevant, alternative X (thus expanding the choice set to {A, B, X}) should not make B preferred to A. In other words, whether A or B is better should not be changed by the availability of X. The IIA axiom simplifies experimental collection of choice data by allowing multinomial choice probabilities to be inferred from binomial choice experiments. It is clear that assumptions *i* and *iii* bear some relation to the invariance principle from measurement theory.

### Mathematics

In conditional logistic regression, none, some, or all of the observations in a choice set may be marked. McFadden’s choice model (discrete choice) is thus a special case of multinomial logistic regression. In the conditional logit (CL) model, the explanatory variables assume different values for each alternative and the impact of level changes is assumed to be constant across alternatives. The model may be summarized as shown below (Formula 4):
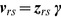
(4)whereby ***v*** are latent values or utilities of individuals choosing health state *s*, ***z*** *_rs_* indicates a vector of *alternative-specific* explanatory variables for individual *r,* and *γ* represents a single vector of unknown regression coefficients. Under the assumptions described above, the probability that health state *s* is chosen is equal to:
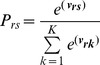
(5a)or,
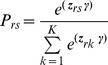
(5b)where, *K* (one *k* has to be set as reference) is the number of alternatives (e.g., health states) in the choice set (e.g., 2 in most DC applications) and *s* is the chosen alternative.

The term multinomial logit (MNL) model refers to a model that generalizes logistic regression by allowing more than two discrete outcomes. It assumes that data are *case-specific*; that is, each independent variable has a single value for each case. Consider an individual choosing among *K* alternatives in a choice set. Let ***x*** *_r_* represent the characteristics of individual *r* and ***β*** *_s_* the regression parameters.
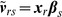
(6)


The probability that individual *r* chooses health states *s* is:
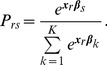
(7)


Both models can be used to analyze an individual’s choice among a set of *K* alternatives. The main difference between the two is that the conventional MNL model focuses on the individual as the unit of analysis and takes the individual’s characteristics as explanatory variables. The CL model, in contrast, focuses on the set of alternatives for each individual, while the explanatory variables are characteristics of those alternatives.

It is possible to combine these two models. Doing so would simultaneously take into account the characteristics of both the alternatives and the individual characteristics, using them as explanatory variables. This combination is sometimes called a conditional MNL or mixed model:
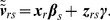
(8)Where 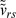 is the value of the alternative *s* assigned by the individual *r*. That value (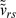) depends on both the alternative characteristics ***x*** and on the individuals’ characteristics ***z***. The probability that individual *r* chooses health states *s* is:



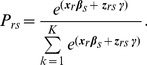
(9)The most commonly applied types of DC models are presented above. A clear distinction is made between models that take an individual’s characteristics as explanatory variables (MNL) and models with explanatory variables for characteristics of alternatives (i.e., health states). In the next section the Rasch model will be explained. It will be shown that this model has a close similarity to the CL model (Equation 5). As the basic data structure underlying the Rasch measurement model should meet the invariance assumption (see ‘measurement principles’), this rules out incorporating elements of the MNL model (Equation 6–9).

## Rasch Model

### Background

The Rasch model – named after the Danish mathematician, statistician, and psychometrician Georg Rasch (1901–1980) – is a probabilistic measurement model. While primarily employed in attainment assessment, it is increasingly used in other areas [49]. Its original setting was the field of reading skills, where it was intended for dichotomous response data (e.g., right/wrong). The field of health outcomes research has shown considerable interest in the topic of Rasch modeling. Recently, attempts have been made to apply the Rasch model to specific HRQoL domains (e.g., pain, depression, mobility) [50], [51].

Rasch did not start from real data but rather from an axiomatic definition of measurement. He formulated a ‘model’, i.e., an equation, fixing the ‘ideal’ relationship between the observation and the amount of the latent trait (i.e., variables that are not directly observed but are inferred, such as utility). At least three features of this relationship should be highlighted. First, the observed response (e.g., pass/yes/agree/right = l, rather than fail/no/disagree/wrong = 0) depends on the difference between only two parameters, the ‘ability’ of the individual and the ‘difficulty’ of the item. No extraneous factors should bias this linear relationship. Second, ‘ability’ and ‘difficulty’ are independent of each other. As stated before, this invariance principle is also a theoretical requirement for measurement in the realm of physics. In his ‘separability theorem’, Rasch demonstrated that his model is the only one that satisfies this requirement. Third, the model is probabilistic: uncertainty surrounds the expected response, which is consistent with the real world situation.

A key element of the Rasch model is that the goal is to construct procedures or operations that provide data that meet the relevant criteria [52]. It should be noted that the Rasch model makes relatively strong assumptions. Nonetheless, if the assumptions hold sufficiently, this measurement model can produce scales (i.e., health-state values) offering a number of advantages over those derived by standard measurement techniques or even contemporary DC models.

Rasch developed the model for dichotomous data. He applied it to response data derived from intelligence and attainment tests, including data collected by the Danish military [53]. It does not confront the respondents with a paired comparison task or a ranking task. Instead, the responses are collected separately (monadic measurement) for a set of items. Versions of the Rasch model are particularly common in psychometrics, the field concerned with the theory and technique of psychological and educational measurement, where they are known as response models. The most important claim of the Rasch model is that due to the mode of collecting response data, in combination with the conditional estimation procedure, the derived measures comply with the three important principles: interval level, unidimensionality, and invariance. Because it uses a specific mechanism (see explanation below), the application of the Rasch model is sometimes referred to as fundamental or objective measurement.

### Measurement Model

The Rasch model is a mathematical function that relates the probability of a (correct) response on an item to characteristics of the person (e.g., ability) and to characteristics of the item (e.g., difficulty). For quantifying health states, this model would relate the probability of a response on a health state to characteristics of an individual (e.g., own health status) and to characteristics of given health states (e.g., severity).

**Figure 1:**
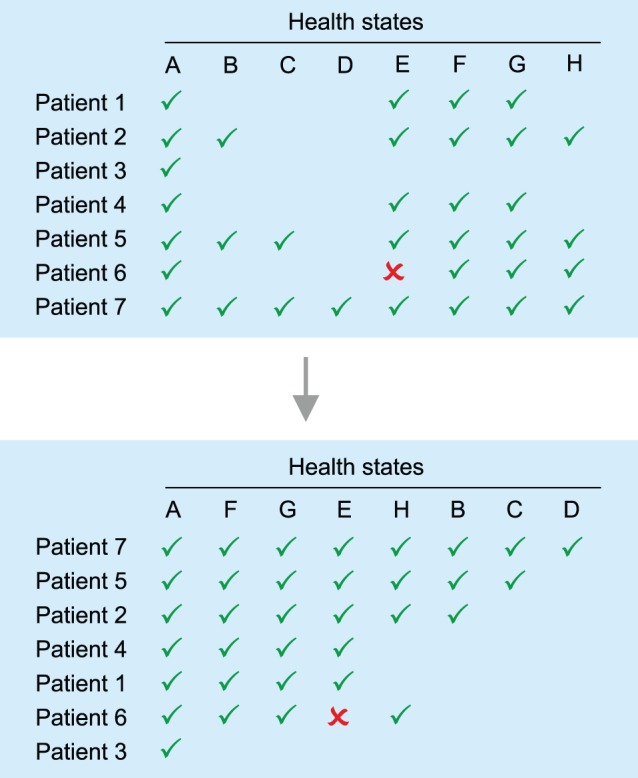
Schematic representation of Guttman/Rasch data structure. Representation of the raw data (top) and after sorting of the columns (health states) and the rows (patients) in order to arrive at the hierarchical Guttman/Rasch data structures (the check mark indicates that this health state is preferred over the next health state, the cross mark indicates a misfit) (from: [33]).

The data structure required by the Rasch model is identical to that of another response model, namely Guttmann scaling, which had been developed independently at an earlier stage [54]. Whereas the Guttmann model is deterministic, the Rasch model is probabilistic. The key to Rasch scaling is in the analysis. Figure 1 (top) shows the responses of 7 patients on 8 health states (A–H). Subsequently, this matrix is sorted so that patients who agree that all health states are preferred over their own health state are listed at the top, and patients agreeing with fewer are at the bottom. For patients with equal number of agreements, the health states are sorted from left to right from states that most agreed to, to states that fewest agreed to. To obtain the specific structure of the data for Rasch analysis the respondents (their own health status) must be distributed over the whole unidimensional scale. Thus, a sample clustered at only one location on the scale (e.g., all healthy people) is not conducive to good estimations of the model. Moreover, the Rasch model can be seen as a practical realization of conjoint measurement (axiomatic theory for quantification of multiple rank-based attributes) with an underlying stochastic structure [55]. For this and other reasons, many scientists consider the Rasch model as the preeminent means to measure subjective phenomena ‘objectively’.

Extensions of the Rasch model have been developed independently and simultaneously; these are known as item response theory (IRT) models [56]. The extensions differ from the original model in the sense that they have a parameter to express the discrimination of an item (the degree to which the item discriminates between persons in different regions on the latent continuum). These IRT models relax to some extent the strict requirements for responses (e.g., data) posed by the Rasch model. But IRT models do not possess the specific fundamental measurement property of the Rasch model and therefore do not necessarily produce cardinal measures [25], [53].

### Mathematics

In the Rasch model for dichotomous data, the probability that the outcome is correct (or that one health state is better than another) is given by:
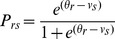
(10)where *θ_r_* identifies the health status *θ* of the person, and *v_s_* refers to the state *s* (In many textbooks, the notation of the Rasch model and other item response theory models is slightly different). By an interactive conditional maximum likelihood estimation approach, an estimate *v_s_ – v_t_* is obtained without involvement of *θ,* which is a special feature of the Rasch model. Formula 10 can be rewritten as:



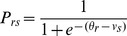
(11)The invariance of measurement principle has two implications for the Rasch model. First, estimates of individual characteristics (person parameter *θ*; i.e., health status) as measured by the instrument are comparable regardless of which health states are included in the instrument. Second, estimates of the position (i.e., severity) of the health states (item parameter *v*) on the scale of the instrument are comparable regardless of the selection of the sample of respondents. This is true as long as the sample reflects the broad spectrum of the scale.

## The Multi-Attribute Preference Response Model

**Figure 2:**
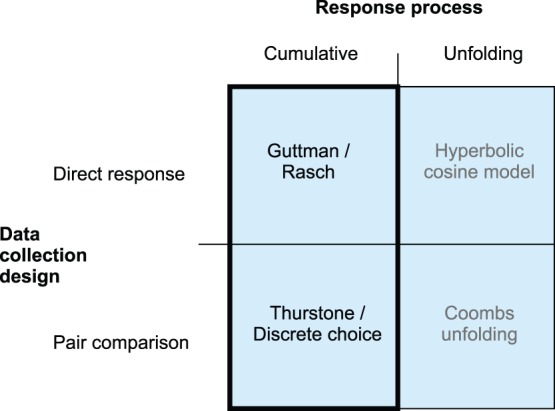
Data collection designs and response processes in measurement models. Schematic representation of the different data collection designs in combination with the specific response process of these designs and the appropriate measurement models for these four combinations (combination of discrete choice model and Rasch model, block bounded by thick line is multi-attribute preference response model).

By incorporating the key response mechanism of the Rasch model into the DC framework, a new and advanced health-status measurement model can be obtained. The strength of the DC models (their capacity not only to quantify health states but also to estimate a value function) can be combined with the strength of the Rasch model (individual patients are given responses to realistic and understandable health descriptions). In principle, such a new model should also encompass the desirable measurement features of the fundamental Rasch model (Figure 2). Moreover, the specific response mode of the Rasch model (patient’s own health state versus other related health states) will largely prevent any adaptation effects. This combination of features from the DC and the Rasch models is referred to as the multi-attribute preference response (MAPR) model. Although there are (subtle) differences between methods from related areas, such as multi-criteria decision analysis (operations research), multi-attribute choice models (decision science), discrete choice models (economics), and the MAPR model, many of the objectives and procedures of these models are the same [57], [58]. However, multi-criteria decision analysis is essentially focused on optimization, the choice models are focused on explaining choice behavior, whereas the MAPR model is focused on measurement (i.e., quantification).

In fact, the Rasch model is closely related to most discrete choice models and their extensions. What makes the Rasch model unique is the person parameter (*θ*) [59], [60]. When dealing only with choice sets consisting of two health states (*s* and *t*), the left part of Formula 5a can be expressed even more succinctly. Dividing the numerator and denominator by the numerator, and recognizing that *e* ^a^/*e* ^b^ = *e* ^(a–b)^, Formula 5a becomes:
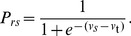
(12)


In this equation it is obvious that the basic formulas for the DC model and the Rasch model differ in only one parameter. The DC model requires two parameters, *v_s_* and *v_t_*. In the Rasch model, one of these parameters is ‘replaced’ by a parameter, *θ*, that represents the location of the respondent (Formula 11). Formula 12 can therefore be rewritten as formula 13. The latter is the basic formula for the MAPR model, created by adding a parameter, ***γ***, for the attributes of the health states:
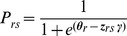
(13)


In this model a set of linear restrictions is imposed on the health-state parameters (*v*) of the Rasch model as, 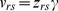. In another setting and approached differently Fischer developed a model that has a close connection with the MAPR model [61], [62].

### Data Collection

**Figure 3:**
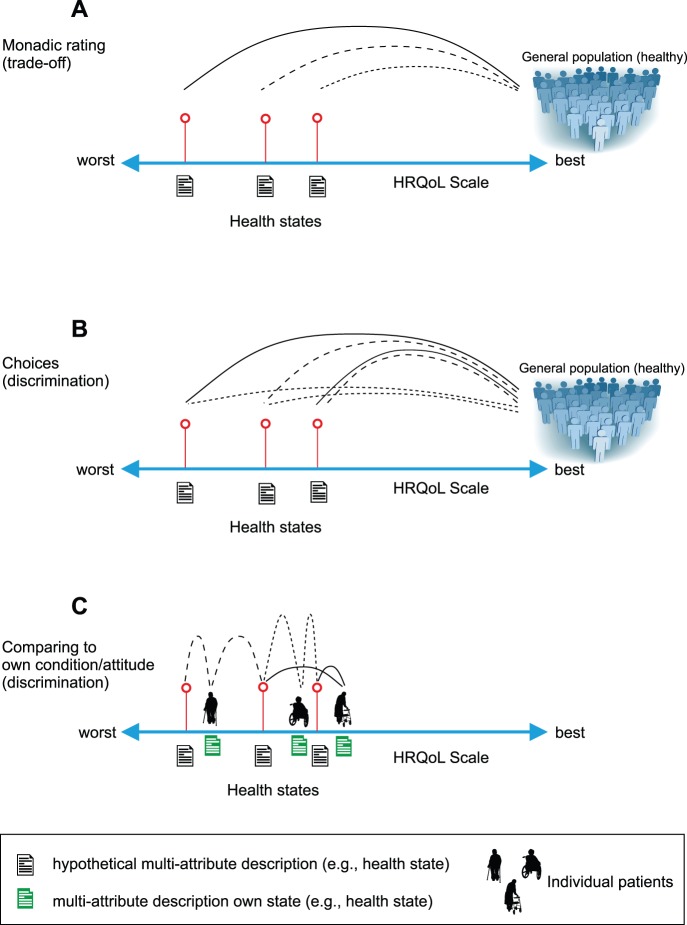
Judgmental tasks used in measurement methods. Schematic representation of the judgmental task for three health states by: A = conventional monadic measurement (SG, TTO) by a sample of the general population; B = conventional discrete choice task (paired comparison) by a sample of the general population; C = multi-attribute preference response model for individual patients (3 patients in this example, each assessing 2 nearby located health states).

**Figure 4:**
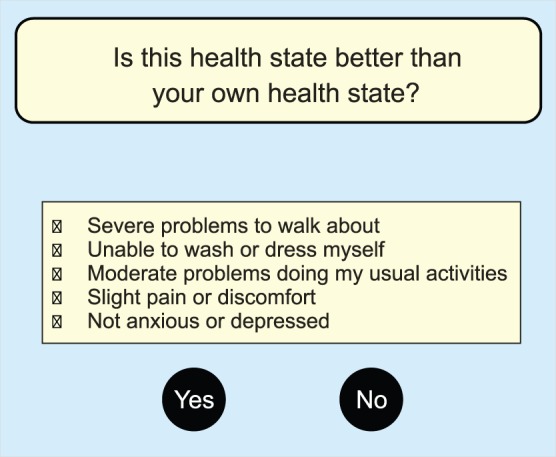
Response task MAPR model. Example of a response task under the multi-attribute preference response (MAPR) model (based on EQ-5D description).

As noted, the Rasch model demands a specific data structure that is essentially different from that of the DC model. The implication is that data have to be derived by new and innovative response tasks. Judgments are required from a heterogeneous sample of people in various health conditions. This means that respondents should not be a representative sample of the general population. Instead, they should be patients who are currently experiencing one of the health states on the continuum from worst to best health status. The reason for this can be seen in Figure 3 that shows graphically the judgmental task for the conventional valuation method TTO (A), the DC task (B), and the MAPR model (C). For the former two a sample of the general population has to assess a pool of states, which is done for the TTO state by state and in the discrete choice task for pairs of states. As the generally population will mainly consist of healthy people they are positioned on the right side on the HRQoL scale. In the MAPR model this is different. Based on the initial classification of their own health status each individual patient is assessing a pool of health (in this example only 2) states that are located in the region of their own HRQoL status. So, patients respond to hypothetical health states by comparing these health states with their own health condition. For example: “Is this health state better than your own health state?” (Figure 4). The conditional (multinomial) logit model will then become similar to the Rasch model. This will occur when the following criteria are met: each comparison consists of two health states, one being the patient’s own state; and the patient’s own health state is considered as a separate parameter in the conditional estimation procedure.

The central mechanism in the MAPR model is that the response task is performed in two distinct stages. First, the classification of the individual’s health status according to the set of health attributes generates a value for the description of that state (based on the underlying value function). In the next step – to which not all respondents has to continue if the value function has reached a certain stage of predictive precision – individuals are confronted with a set of health states that cover the range from severe to mild or a set of other almost ‘equivalent’ states (see below: pivot designs) that are compared to their own state that is determined at the first stage (such a comparison may be more easy than under DC). The information generated in this part is used to arrive at a more precise value function. Of course, this iterative mechanism only operates properly after a large number of individuals have gone through both stages. So, there is clearly an initiation stage in which patients are performing the judgmental tasks to feed the statistical part of the MAPR model. At this stage the value of the health condition of the patients themselves cannot be (precisely) estimated. It will take probably 1000 or more patients to conduct the inception of the MAPR model and arrive at a functional routine. A relatively large number of respondents is required as the MAPR model has to estimate health-state parameters and patient parameters, all based on binary data.

As a first exploration of the MAPR model, we may start with the most basic variant in which an existing health-status classification system is used (e.g., EQ-5D). First, patients (representing the whole continuum from bad to mild health conditions) classify their own health condition on the basis of the EQ-5D classification. Then they judge a fixed set (say 20) of EQ-5D health states (representing the whole continuum from bad to mild states).

### Pivot Designs

In standard DC studies, all respondents face the same choice situations (e.g., pair of health states) or a selection of them. While this is also possible for the MAPR model, it is not an efficient approach. Rather than presenting patients with a predetermined set of health states, it would be better to frame the choice task within an existing decision context, namely a situation pertaining to an individual person. This strategy makes use of pivot designs. First, the respondents classify their own health status according to a standardized system (e.g., EQ-5D, HUI). Then they are shown alternatives with attribute levels that deviate slightly from their own levels. Several approaches for finding efficient pivot designs have been developed. Upon comparison, the most attractive one proves to be based on the individual’s responses [63]. Because this approach entails a separate design for each respondent, it should also yield the highest efficiency. It is well known that a test item provides the most information about a respondent when its severity is roughly the same as that of the person’s health status. Such an approach requires a computer adaptive testing environment (e.g., an internet survey). An efficient design is generated interactively and is therefore well suited to the proposed MAPR model for quantifying health states.

### Individual Choices of Health Domains

The MAPR model can even be extended to cover a large set of candidate domains. An individual patient could then select the ones most relevant to his or her assessment (far more than the traditional four to nine domains in existing instruments). Recently, specific solutions (partial profile choice designs) for this situation have been introduced [64]. In such an extended variant of the MAPR, the precision of the underlying value function will increase step by step. The reason is that individuals then use more and more attributes when comparing their own health status with other health states. Of course, the overall estimation and convergence of such a MAPR model requires substantial input from the patients. At present, many aspects of these partial profile designs remain to be investigated in more detail, in particular in the setting of health-state valuation – a challenging task. For example, one of the elementary assumptions in applying this type of model is that all the attributes (health domains) that are not part of individual judgmental tasks are to be set to ‘0′ (no value). Studies in other areas have shown that this assumption does not always hold [65].

## Discussion

This article presents a new measurement approach, the multi-attribute preference response model (MAPR), to quantify subjective elements of health. The MAPR model can be considered as an adaptation of the standard discrete choice model or as an extension of the standard Rasch model. This paper also shows that seemingly different models are often very similar. Conventional valuation techniques are known to have many problems, such as adaptation, discounting, context, reference point, and other effects [66]. With the MAPR model, unwanted mechanisms affecting valuations of health states may be largely eliminated.

From a theoretical and practical point of view, both the MAPR and the DC models are more attractive than TTO and other conventional valuation techniques. In the MAPR and DC models, the judgmental task and the analysis are executed within one unifying framework. This is different for the TTO, where the valuation technique and the statistical model to estimate a value function are distinct (e.g., using different regression techniques, different coding of the parameters). The strength of the DC and MAPR models is that the derived values relate to one particular aspect: the attractiveness of a health state. Such measures are not confounded by aspects such as time preference (duration of time frame) or the choice for an indifference procedure, nor are there difficulties in quantifying states worse than dead. It is sometimes said that, unlike the TTO technique, most of the present choice models to quantify health states do not specify the duration of the states. However, as the two states in each pair are rather similar in these type of studies, including a duration statement would hardly affect the responses. Furthermore, discrimination is a basic operation of judgment and a means of generating knowledge. The core activity of measurement in probabilistic choice models is to compare two or more entities in such a way that the data yields compelling information on individuals’ preferences, thereby imitating choices in daily life.

The Rasch model occupies a special position in the field of subjective measurement, although mathematically it is closely related to item response theory. Broadly speaking there are two general schools of thought, each known for its particular approach. When the response data satisfy the conditions required by these mathematical models, the estimates derived from the models are considered robust. When the data do not fit the chosen model, two lines of inquiry are possible. In essence, when the data do not fit a given model, the IRT approach is to find a mathematical model that best fits the observed item-response data. By contrast, the Rasch measurement approach is to find data that better fit the Rasch model. Thus, it follows that proponents of IRT use a family of item-response models, while proponents of Rasch measurement use only one model (Rasch model).

A major limitation of the MAPR model, as with any probabilistic choice model, is that it produces relative positions of all health states on the latent scale [30], [67]. For the estimation of DALYs and QALYs, however, those values need to be on the ‘dead’– ‘full-health’ scale. If the MAPR model is used to value health, a way must be found to link the position of ‘dead’ with the derived values. A similar solution may also be relevant to locate the position of the best (dominant) health state of a multi-attribute classification system to ‘full health’. A strategy for rescaling DC values may be to anchor them on values obtained for the best health state and for ‘dead’ using other valuation techniques, such as TTO or SG. However, the rationale for this approach is unclear. Part of the motivation for adopting probabilistic choice models as potential candidates to produce health-state values lies in the limitations of existing valuation techniques. Alternatively, the judgmental format may be set up in such a way that the derived health-state values can be related to the value of the state ‘dead’. A simple manner to achieve this seems to involve making designs in which respondents are presented with at least one bad health state at a time and asked if they consider it better or worse than being dead. The procedure has been demonstrated by McCabe et al. [37] and Salomon [31]. These authors mixed the state ‘dead’ in with the choice set as a health state, so that a parameter for the state ‘dead’ is estimated as part of the model. To investigate whether or not this strategy would produce health-state values with acceptably low bias, it will be necessary to draw comparisons with values obtained using alternative measurement methods. A problem associated with including ‘dead’ as part of the choice set is that proper estimation of values seems only possible if almost all respondents consider some health states to be worse than dead. Otherwise, the estimated parameters of the model are likely to be biased [68]. Another approach could employ models that are suitable for dealing with dominant health states (the best health states in a multi-attribute system) to calibrate the metric distances in this region [69]. One example would be to apply multidimensional scaling [70]. In sum, it is hard to say beforehand which approach to deriving DALY/QALY values that are anchored to ‘dead’ would produce valuable and effective results for the MAPR model (DC models have the same problems). Theoretically competent construction of MAPR models and subsequent experimentation with these models would therefore be required to see how these difficulties could be resolved in a particular situation.

Several elements related to the MAPR model must be investigated empirically to confirm certain assumptions and explore potential limitations. In particular, the Guttman/Rasch structure of the data has to be proven. As the data collection for the MAPR model is done in patient groups, suboptimal response data may be resulting from interpretation problems of the health domains and their levels, from using cognitive shortcuts, from irrational choice behavior, and other factors.

One of the crucial elements that has to be decided on before any measurement of health status can take place is the selection of the health domains to conceptualize health-state descriptions. An overwhelming number of health-status or HRQoL instruments are available. Each has been developed with a particular concept in mind, resulting in instruments with a specific depth (basic or subordinate units of information; for example, physical function, self-care, bathing) and breadth (e.g., physical function, emotional function, pain, cognitive ability). However, all current descriptive and preference-based health-status instruments are based on a predetermined restricted set of health domains. This common denominator has certain drawbacks. For example, for some patients or diseases, the predetermined set may miss some crucial health domains. Extending it to create a very broad set is not feasible for several reasons, both practical and analytical. In light of information theory, it is clear that a limited set of domains may deliver enough detailed information to adequately describe the health status of a person if these domains are well selected and reflect the domains most relevant to this specific person.

Therefore, an advantageous property of the MAPR model is that it can overcome the limitation of the restricted set of health domains, which is a drawback of the existing preference-based health-status systems (e.g., HUI, EQ-5D). In many instances, these instruments prove to be insensitive or invalid, due to their restricted and fixed set of health attributes. The MAPR model, in contrast, can be extended to include a large set (40–120) of candidate domains from which an individual patient can select a few (5–7), namely the most relevant ones. A large number of individual responses would be needed to collect enough data. As there is no shortage of patients, the estimation part of this model from a practical point of view is not too challenging [64]. Of course, the statistical routines for such an approach have to be developed, and ultimately, empirical research must prove the premise of such an extended MAPR model. An instrument that shows some resemblance to the measurement strategy of the MAPR model, in which the content is derived from individual patients, is the SEIQoL [71]. Although it is not embedded in a formal measurement model, this instrument permits individuals to select and value the health domains that are important to them in their HRQoL assessment. The EuroQol Group is planning to develop special ‘bolt-ons’ (comprising additional health attributes) that will be added to the existing five health attributes of the EuroQol-5D system [72]. Yet, the analytical integration of these bolt-ons does not seem to be part of an overarching framework. The Group has indicated that in the end the maximum number of predetermined attributes will be seven instead of five.

Existing multi-attribute preference-based health-status systems are conceptualized as having two stages: one encompasses the valuation study to derive the value function; the second comprises an application stage, in which the health status of individuals is determined. One of the major strengths of the MAPR model is that it is a continuous process of valuation and application.

Patients may be regarded as the best judges of their own health status. Therefore, it is sensible to defend the position that it is the patient’s judgment that should be elicited. However, it may be the case that values for health states worked out under the MAPR model both for patients and a sample of unaffected members of the general population are rather similar [19]. Otherwise, empirical MAPR head-to-head studies may reveal that responses from the general population are evenly valid, except maybe for the very worst health states. These type of health states may be under or overestimated by healthy people due to lack of familiarity with the impact of seriously reduced health.

The MAPR model may also be an avenue for developing a health-status measurement instrument that can be used in broader settings (e.g., medical interventions *and* medical care) and in distinctive patient populations (e.g., children, adults, elderly). Another possible extension of the MAPR model would be to combine it with Monte Carlo simulation and then to use this technology in estimating response models within a full Bayesian framework [73], [74]. In principle, the MAPR model may be suitable to measure other unidimensional, subjective phenomena such as well-being, capabilities, and happiness; under certain conditions it might be used in social value judgments (e.g., reimbursement decisions). For the overall quantification of quality in general the MAPR model may also be beneficial [75]. Our group is currently working on different variants of the MAPR model and the related estimation functions. In addition, the first empirical patient studies are planned.

## Conclusions

Incorporating the basic elements of the Rasch model into the DC framework (or vice versa) produces an advanced model with fundamental measurement characteristics: the multi-attribute preference response (MAPR) model. This new patient-reported outcome measurement model is more coherent than the conventional valuation methods and has a profound connection to measurement theories. The MAPR model can be applied to a wide range of research problems. Specifically, if extended with self-selection of relevant health attributes for the individual patient, this model will be more valid than existing valuation techniques.
